# Force-induced chemical reactions on the metal centre in a single metalloprotein molecule

**DOI:** 10.1038/ncomms8569

**Published:** 2015-06-25

**Authors:** Peng Zheng, Guilherme M. Arantes, Martin J. Field, Hongbin Li

**Affiliations:** 1 Department of Chemistry, University of British Columbia, Vancouver, British Columbia, Canada V6T 1Z1; 2 School of Chemistry and Chemical Engineering, Nanjing University, Nanjing 210063, P. R. China; 3 Departamento de Bioquímica, Instituto de Química, Universidade de São Paulo, Avenue Lineu Prestes 748, São Paulo SP 05508-900, Brazil; 4 Institut de Biologie Structurale (IBS) Jean-Pierre Ebel, CEA/CNRS/Universite Joseph Fourier, 71 Avenue des Martyrs, CS 10090, Grenoble 9 38044, France

## Abstract

Metalloproteins play indispensable roles in biology owing to the versatile chemical reactivity of metal centres. However, studying their reactivity in many metalloproteins is challenging, as protein three-dimensional structure encloses labile metal centres, thus limiting their access to reactants and impeding direct measurements. Here we demonstrate the use of single-molecule atomic force microscopy to induce partial unfolding to expose metal centres in metalloproteins to aqueous solution, thus allowing for studying their chemical reactivity in aqueous solution for the first time. As a proof-of-principle, we demonstrate two chemical reactions for the FeS_4_ centre in rubredoxin: electrophilic protonation and nucleophilic ligand substitution. Our results show that protonation and ligand substitution result in mechanical destabilization of the FeS_4_ centre. Quantum chemical calculations corroborated experimental results and revealed detailed reaction mechanisms. We anticipate that this novel approach will provide insights into chemical reactivity of metal centres in metalloproteins under biologically more relevant conditions.

## Abstract (web summary)

The investigation of the chemical reactivity of metal centres in metalloproteins in aqueous solution is challenging. Here, the authors demonstrate the use of single molecule force spectroscopy to study the chemical reactivity of the iron-sulfur centre in rubredoxin in aqueous solution.

## Graphical abstract

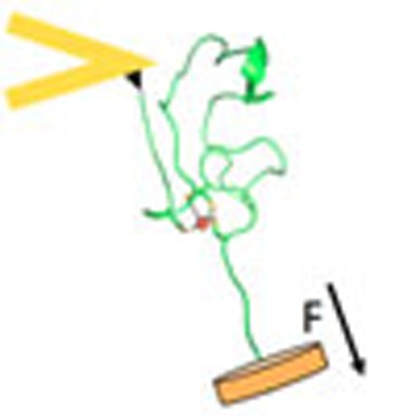

Metalloproteins are ubiquitous in nature and play indispensable roles in a wide range of cellular processes^1^,^2^. Owing to their versatile chemical reactivity, incorporated metal ions add functionality to proteins and help to catalyse some of the most difficult reactions in nature^3^. Because of this, the investigation of metal centre reactivity is of great importance for understanding metalloprotein function and mechanism of action. In many metalloproteins, labile metal centres are often enclosed and protected by the three-dimensional (3D) structure of the polypeptide chain, limiting access to reactants and making it difficult to study their chemical reactivity in aqueous environment.

To overcome the challenges arising from protein structures, synthetic analogue strategies have been developed. These strategies involve the synthesis of metal complexes that mimic the natural metal centres in proteins, but lack a 3D protein scaffold. For example, FeS clusters from iron–sulfur proteins were some of the earliest types of metal centres that were synthesized, and the study of such metal centres has provided much valuable structural and functional information^4^,^5^. The first kinetic study of a ligand substitution reaction on a synthetic cluster Fe_4_S_4_(SR)_4_^2−^,which serves as the analogue for the active site of ferredoxin, was accomplished in the early 1970s (refs 6, 7). In addition, protonation chemistry on the cluster has also been widely demonstrated^8^. These studies revealed the versatile reactivity of the FeS cluster in iron–sulfur proteins, suggesting that such reactivity may be important for the catalytic functions of some metalloproteins *in vivo*^9^, such as the nitrogenases and hydrogenases.

By contrast, direct demonstration of the reactivity of metalloprotein FeS centres in aqueous solution has been challenging, in part because of the fact that metal centres are often buried and inaccessible to the aqueous environment. The addition of chemical denaturants, which disrupts the protein structure and exposes metal centres to the solution, is often used to investigate the reactivity of FeS centres in metalloproteins in aqueous solution^10^,^11^. However, there is growing evidence that the protein structure close to the metal centre can significantly influence and regulate the reactivity of the metal centre, making the use of chemical denaturants potentially problematic^3^,^12^. As a result, it is necessary to develop alternative and complementary methods to study the properties of such metal centres by accessing the metal centre while maintaining protein native structure as much as possible.

Single-molecule force spectroscopy has evolved into a powerful technique for investigating force-induced conformational changes in macromolecules and chemical reactions through the application of a stretching force with picoNewton precision to individual molecules^13^,^14^,^15^,^16^,^17^,^18^,^19^,^20^. In particular, single-molecule force spectroscopy has enabled the investigation of protein unfolding/folding reactions under a stretching force in great detail. The experimental conditions offered by force spectroscopy closely mimic the physiological environment of a wide range of proteins that are subjected to a stretching force in a variety of biological processes, including muscle contraction^21^, protein translocation^22^ and protein proteasomal degradation^23^. Such environments are also relevant for some metalloproteins. For example, metalloprotein superoxide dismutase 1 is known to follow a proteasomal degradation pathway and its forced unfolding is an important step in its degradation and the disassembly of its metal centres^24^.

Our previous studies have shown that single-molecule atomic force microscopy (AFM) can be used to investigate the unfolding/folding mechanism of metalloproteins and the disassembly of metal centres^25^,^26^,^27^,^28^,^29^. In a force spectroscopy experiment, a protein is stretched from two specific residues and can undergo force-induced unfolding. Weak interactions/bonds can be ruptured along the unfolding pathway^13^,^16^,^17^,^20^. Rubredoxin is the first metalloprotein that has been studied in detail using single-molecule AFM. Our results showed that the FeS_4_ centre is ruptured as the small iron–sulfur protein rubredoxin is unfolded^26^,^27^. Subsequent molecular simulations and quantum chemical (QC) calculations provided a detailed description of rubredoxin unfolding and the rupture mechanism of ferric–thiolate bonds^30^. Our results showed that specific residues (1–5 and 41–53) outside of the metal centre can be first unfolded and extended before rupture of the FeS_4_ centre. As a result, the FeS_4_ centre can be exposed to the aqueous environment, while the protein structure between residues 5 and 41 remains largely intact^25^,^28^. We reasoned that the FeS_4_ centre exposed in this way could readily access chemical reactants in solution and participate directly in chemical reactions within the bulk solution, while the rest of the protein structure remains folded.

Here we use the well-characterized rubredoxin as a model system to demonstrate the utility of AFM to probe the chemical reactivity of metal centre in metalloproteins in aqueous environments. We investigate two different chemical reactions at the iron–sulfur centre in rubredoxin: electrophilic protonation chemistry and nucleophilic ligand substitution. We demonstrate that protonation of the FeS_4_ centre occurs when the metal centre is exposed as a result of the force-induced partial unfolding of rubredoxin. We observed that the rupture force and bond lifetime of ferric–thiolate bonds in the FeS_4_ centre is significantly decreased in acidic solutions as a result of protonation of the FeS_4_ centre. In addition, we found that substitution with thiocyanate (SCN^−^), a weaker nucleophile compared with thiolate for ferric ion, is facilitated by the application of a mechanical stretching force. The mechanical rupture rate of ferric–thiolate bonds in the presence of SCN^−^ is linearly dependent on SCN^−^ concentration. Further details into the molecular mechanisms and distinctive features of these reactions in the FeS_4_ centre in rubredoxin were elucidated using QC calculations. By combining single-molecule force spectroscopy with QC calculations, our work represents a new approach towards studying the chemical reactivity of metal centres in metalloproteins, one that we anticipate will provide insights into the chemical reactivity of metal centres in aqueous solution under biologically relevant conditions.

## Results

### Protonation chemistry on the FeS_4_ centre of rubredoxin

**Figure 1:**
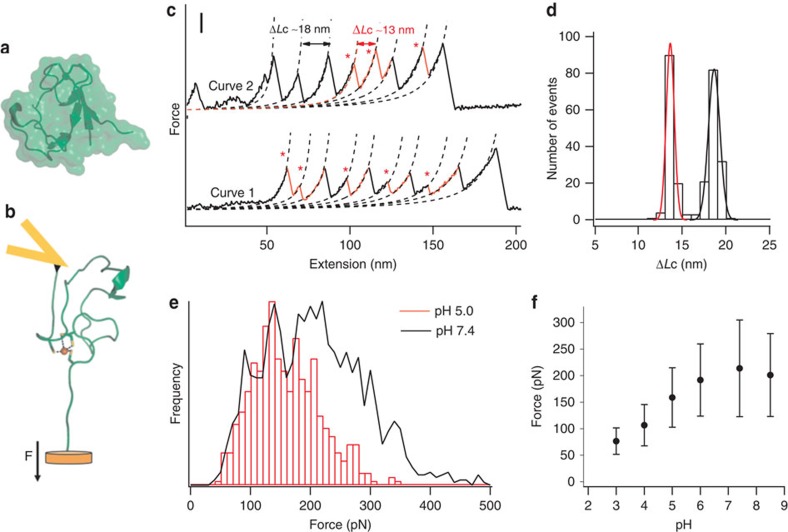
Partial unfolding of rubredoxin by force exposes the FeS_4_ centre and allows for protonation of the metal site. (**a**) The structure of rubredoxin, depicted in both cartoon and surface models, shows that the FeS_4_ centre is buried within the protein structure. (**b**) A schematic showing how rubredoxin can partially unfold during stretching in the AFM experiments, leading to the exposure of the FeS_4_ centre to the aqueous environment. (**c**) Typical force-extension curves of stretching polyprotein (RD-GB1)_*n*_ at a pH of 5.0 (curve 1) and a pH of 7.4 (curve 2). Both curves show force peaks corresponding to the rupture of the FeS_4_ centre, which are characterized by ΔLc of 13 nm and is indicated by *, and unfolding of the fingerprint domain GB1 (ΔLc of 18 nm). Scale bar for the *y* axis, 100 pN. (**d**) A histogram of ΔLc of (RD-GB1)_*n*_ at pH 5 shows two distributions: one is centred at ∼13 nm, and the other one is at ∼18 nm. Gaussian fits (solid lines) to the experimental data measure a ΔLc of 13.2±0.5 nm for rubredoxin and 18.1±0.6 nm for GB1. (**e**) Rupture force histograms of rubredoxin at pH 7.4 (in black) and pH 5 (in red). These histograms clearly show that the rupture force of the FeS_4_ centre decreases at lower pH. (**f**) The rupture force of ferric–thiolate bonds decreases as the pH decreases from 7.4 to 3. The error corresponds to the s.d.

Rubredoxin is the simplest iron–sulfur protein found in nature, with only 53 residues enclosing the FeS_4_ centre active site^31^,^32^, and its mechanical unfolding and the disassembly of the FeS_4_ centre have been characterized in detail using single-molecule AFM techniques and QC calculations^25^,^26^,^27^,^28^,^29^,^30^. In the FeS_4_ centre, ferric–thiolate bonds between the sulfur atoms of four cysteine residues coordinate the ferric ion. As shown in Fig. 1a, the metal ion is buried inside the protein, making it difficult to access by exogenous reactants in solution. Although many iron–sulfur clusters participate in protonation chemistry^8^, such reactions cannot be detected by simply putting the protein in mildly acidic solutions. For example, the FeS_4_ centre in rubredoxin is stable even at pH 2 (10 mM H^+^) at room temperature^33^. However, force-induced partial unfolding of the protein should lead to the exposure of the FeS_4_ to aqueous environment (Fig. 1b), making it feasible to study the chemical reactivity of the FeS_4_ centre in aqueous environments.

To directly investigate the chemical reactivity of the FeS_4_ centre in rubredoxin, we constructed a polyprotein chimera (RD-GB1)_*n*_ to use in single-molecule AFM experiments, where RD represents rubredoxin. It incorporates the well-studied GB1 domain as a fingerprint to help identify single-molecule stretching events^34^. In addition, the GB1 domain is a non-metalloprotein whose mechanical stability is largely unaffected by pH and ligand substitution, and is thus a perfect control and internal force caliper for investigating the effect of pH on the rupture of FeS_4_ centre in rubredoxin in our AFM experiments^35^. Stretching the polyprotein (RD-GB1)_*n*_ results in force-extension curves with a characteristic sawtooth pattern, in which each individual sawtooth corresponds to the mechanical unfolding of an individual domain. Unfolding events with a contour length increment Δ*L*c of 18 nm correspond to GB1 unfolding events, while those with a Δ*L*c of 13 nm correspond to the complete unfolding of rubredoxin and the rupture of its FeS_4_ centre (Fig. 1c). The unfolding force for rubredoxin corresponds mainly to the force required to rupture its FeS_4_ centre, as apo-rubredoxin unfolds at forces that are below the detection limit (∼20 pN) of our AFM^26^.

Many residues in rubredoxin, including the sulfur atoms involved in the ferric–thiolate bonds, can easily become protonated. Since apo-rubredoxin unfolds at forces below 20 pN under acidic conditions (data not shown), which is similar to that under neutral conditions, changes of unfolding force of holo-rubredoxin under acidic conditions will reflect the effect of protonation of residues that directly affect the mechanical strength of the FeS_4_ centre. In fact, protonation of sulfur atoms will result in Fe–S bond weakening and an acceleration of thiolate bond rupture. Thus, monitoring the force at which the FeS_4_ centre ruptures under acidic conditions should provide valuable information about the protonation state of the FeS_4_ centre.

Stretching the polyprotein (RD-GB1)_*n*_ in the presence of 0.01 mM H^+^ (pH 5) resulted in typical force-extension curves with a sawtooth pattern appearance (Fig. 1c, curve 1). Fitting the experimental data to the worm-like chain model of polymer elasticity^36^ revealed that the unfolding force peaks show two distinct contour length increments. This is similar to the results of previous experiments performed at neutral pH (Fig. 1c, curve 2)^26^. The Δ*L*c histogram clearly shows these two distributions (Fig. 1d). In addition, the average unfolding force of the GB1 domain is similar under the two conditions, specifically 194±48 pN (average±s.d., *n*=284, pH 7.4) and 190±49pN (*n*=441, pH 5). This clearly shows that protonation does not affect the unfolding of GB1. In contrast to this, the rupture of the FeS_4_ centre was significantly affected by a decrease in pH. The rupture force of the ferric–thiolate bonds at pH 5 was considerably lower (160±60 pN (*n*=500)) than that measured at neutral pH (211±86 pN, *n*=1421; Fig. 1c). A clear shift towards lower force is also observed within the rupture force histogram (Fig. 1e). Figure 1f shows the rupture force of ferric–thiolate bonds as a function of pH, from which it is evident that the mechanical bond strength of the ferric–thiolate bond is significantly weakened at an acidic pH when compared with a neutral pH. This change in the ferric–thiolate bond rupture force is likely caused by protonation of the metal site at acidic pH.

To obtain further insights about how protonation affects the ferric–thiolate bond rupture, we performed stretching experiments at different pulling speeds (Supplementary Fig. 1) and measured the pulling-speed dependence of these rupture forces. Using a well-established Monte Carlo procedure^37^, we estimated the ferric–thiolate bond dissociation rate constant under zero force, and the distance between the bound state and the transition state, Δ*x*_u_. An acidic pH significantly increased the Δ*x*_u_ when compared with the bond rupture of ferric–thiolate bonds at neutral pH (from 0.11 nm at neutral pH to 0.17 nm at acidic pH). This result corroborates our assertion that protonation likely occurs on the thiolate ligand and changes the rupture mechanism of the FeS_4_ centre at acidic pH. In addition, the spontaneous dissociation rate, *α*_0_, increases with a decrease in pH (an increase in [H^+^]), indicating how protonation affects the ferric–thiolate bond rupture process. This result indicates that the sulfur atom of the ferric–thiolate bond is indeed protonated during the rubredoxin unfolding process, demonstrating the chemical reactivity of sulfur atoms in the FeS_4_ centre (Supplementary Fig. 2).

### Ligand substitution on the rubredoxin FeS_4_ centre

**Figure 2:**
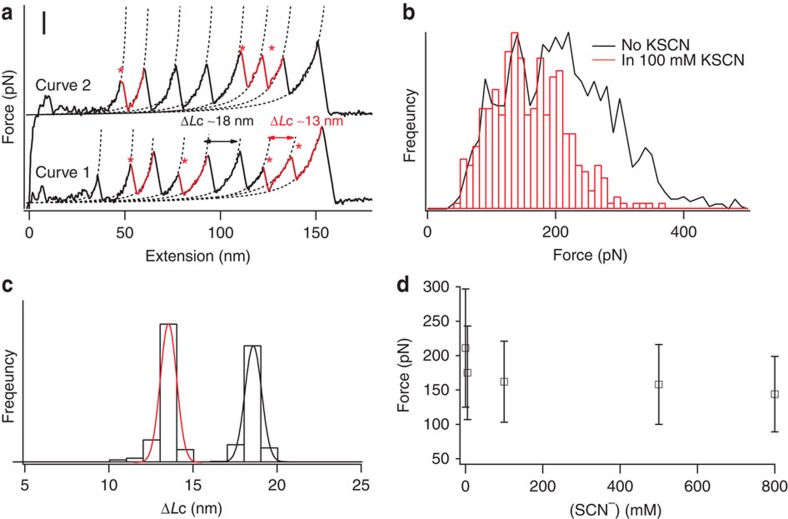
Mechanical unfolding of rubredoxin at various KSCN concentrations. (**a**) Typical force-extension curves of stretching polyprotein (RD-GB1)_*n*_ in 100 mM KSCN (curve 1) and in the absence of KSCN (curve 2). Both curves show force peaks corresponding to the rupture of the FeS_4_ centre, which are characterized by ΔLc of 13 nm and indicated by *, and unfolding of the fingerprotein domain GB1 (ΔLc of 18 nm). Scale bar for the *y* axis is 100 pN. (**b**) Rupture force histograms of rubredoxin in the presence of 100 mM KSCN (in black) and in Tris (in red). These histograms clearly show that the rupture force of the FeS_4_ centre decreases in the presence of 100 mM KSCN. (**c**) The histogram of ΔLc of (RD-GB1)_*n*_ in the presence of 100 mM KSCN shows two distributions: one is centred at ∼13 nm and the other one is at ∼18 nm. Gaussian fits (solid lines) to the experimental data measure a ΔLc of 12.9±0.5 nm for rubredoxin and 18.0±0.5 nm for GB1. (**d**) The rupture force of ferric–thiolate bonds decreases as the KSCN concentration increases.

Force can be used not only to probe the consequence of protonation of the ferric–thiolate bonds, which is a common reaction for iron–sulfur clusters, but may also be exploited to mechanically activate the metal–ligand bond, thus making a suite of otherwise difficult reactions possible. To examine this possibility, we carried out a ligand substitution reaction on the FeS_4_ centre in rubredoxin using SCN^−^, which is a weaker nucleophilic ligand than thiolate and so does not normally substitute for thiolate at the FeS_4_ centre. We carried out force-extension measurements of rubredoxin in the presence of SCN^−^ (Fig 2). As shown in Figs 2a,b, the ferric–thiolate bond rupture force decreases in the presence of SCN^−^, suggesting that SCN^−^ weakens the ferric–thiolate bond. Moreover, this weakening effect is dependent on the concentration of SCN^−^, where increasing concentrations of SCN^−^ result in a decrease in bond strength (Fig. 2d). These results clearly indicate that, although SCN^−^ is a weaker nucleophilic ligand, it still affects the mechanical rupture of the FeS_4_ centre. This seeming paradox can be explained if we suppose that stretching forces weaken the FeS_4_ centre to such an extent that SCN^−^ can start to compete with thiolate for the ferric ion. It is worth noting that rubredoxin is much less sensitive to the effect of SCN^−^ than it is to protonation, as a minimum concentration of 5 mM thiocyanate is necessary for an observable effect (compared with a minimum proton concentration of 0.01 mM).

**Figure 3:**
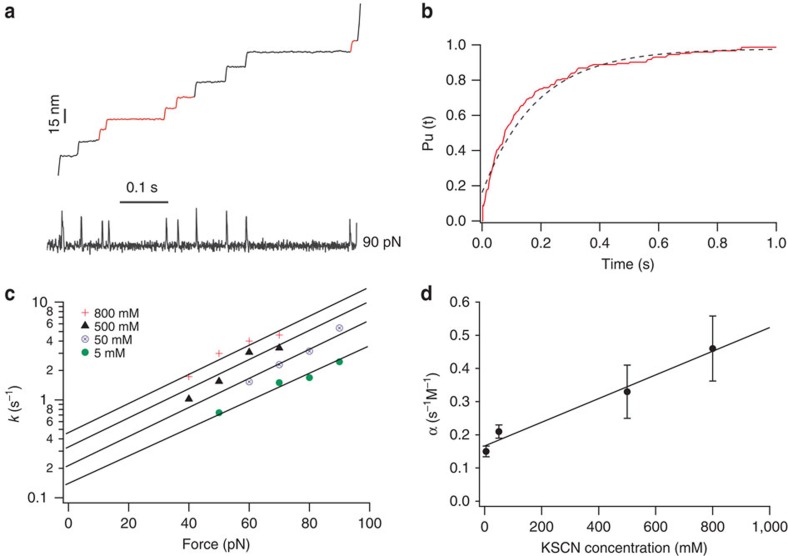
Force clamp experiments characterize the rupture rate of ferric–thiolate bond in the presence of KSCN. (**a**) Representative length-time curves of (rubredoxin-GB1)_*n*_ under a constant force of 90 pN in the presence of 50 mM SCN^−^. Steps coloured in black correspond to the unfolding events of GB1, while those in red correspond to the unfolding event of rubredoxin. (**b**) Average time course of unfolding of rubredoxin obtained by summation and normalization of the rubredoxin portion of 51 curves such as the one in **a**. The unfolding time course of rubredoxin in the presence of 50 mM SCN^−^ can be described by a single exponential (blue dotted line) with a rate constant of 5.39 s^−1^. (**c**) Semi-logarithmic plot of FeS_4_ centre rupture rate as a function of force under different concentrations of KSCN. The solid lines are fits based on the Bell–Evans model, giving the spontaneous rupture rate of FeS_4_ in the presence of KSCN. (**d**) Rupture rate constant of FeS_4_ as a function of KSCN concentration. The solid line is a linear fit to the data. This linear dependence on [KSCN] demonstrates that the SCN^−^ ligand substitution reaction is first order with respect to SCN^−^.

We then carried out force clamp experiments^38^,^39^ to obtain quantitative information about how SCN^−^ affects rupture kinetics. Figure 3a shows a typical extension-time trace of (RD-GB1)_*n*_ in the presence of 50 mM KSCN under a constant stretching force of 90 pN. Normalized ensemble averages of length versus time from 51 molecules (Fig. 3b) can be fit using a single exponential relationship; this was used to measure the ferric–thiolate bond rupture rate in the presence of 50 mN SCN^−^ at a force of 90 pN. By carrying out force clamp experiments at different forces, we found that the logarithm of the rupture rate of ferric–thiolate bonds depends linearly on the pulling force, thereby following force-rupture behaviour predicted by the classic Bell–Evans model (Fig. 3c). Fitting the experimental data to the Bell–Evans model^40^ allowed us to estimate the spontaneous rupture rate of ferric–thiolate bonds at zero force in the presence of a given SCN^−^ concentration. Substitution rates were obtained from a linear fit of the semi-logarithm plot, specifically 0.15, 0.21, 0.33 and 0.46 s^−1^ at KSCN concentrations of 5, 50, 500 and 800 mM, respectively (Fig. 3c). The distance between bound state and mechanical transition state is ∼0.14 nm under each concentration. We found that the rupture rate of ferric–thiolate bonds is linearly dependent on [SCN^−^], suggesting that the rupture of ferric–thiolate bonds in the presence of SCN^−^ is first order with respect to [SCN^−^]. This linear relationship can be described as *r*=*k* × [SCN^−^]+0.15, where *r* is the ferric–thiolate bond dissociation rate in the presence of SCN^−^, *k* (the rate constant) is equal to 0.36 M^−1 ^s^−1^ (Fig. 3d) and the intercept of 0.15 indicates the spontaneous bond dissociation rate of Fe–S bond in the absence of SCN^−^. Ligand substitution reactions are common reactions in synthetic analogues of Fe–S clusters from iron–sulfur proteins, and are often second-order reactions, being first order with respect to both the metal cluster and the competing agent^9^. For example, several ligand substitution reactions on the alkylthiolate tetramer dianion Fe_4_S_4_(SR)_4_^2−^, which is an analogue of the active site of ferredoxin, exhibit rates that are linearly dependent on the concentration of the competing agent R'SH (refs 7, 41, 42, 43). Our results that the SCN^−^ substitution reaction on the ferric–thiolate bond in rubredoxin is first order with respect to SCN^−^ when the pH is kept at 7.4 are in good agreement with studies of similar reactions using inorganic analogues of the FeS cluster from iron–sulfur proteins.

### QC calculations outline the FeS bond cleavage mechanism

Our experimental results on protonation and ligand substitution reactions on the FeS_4_ centre in rubredoxin demonstrate that chemical reactions can be directly monitored in iron–sulfur proteins in aqueous solution in the absence of chemical denaturants, where the protein is only partially unfolded by a stretching force and the protein structure surrounding the metal centre is still largely present.

**Figure 4:**
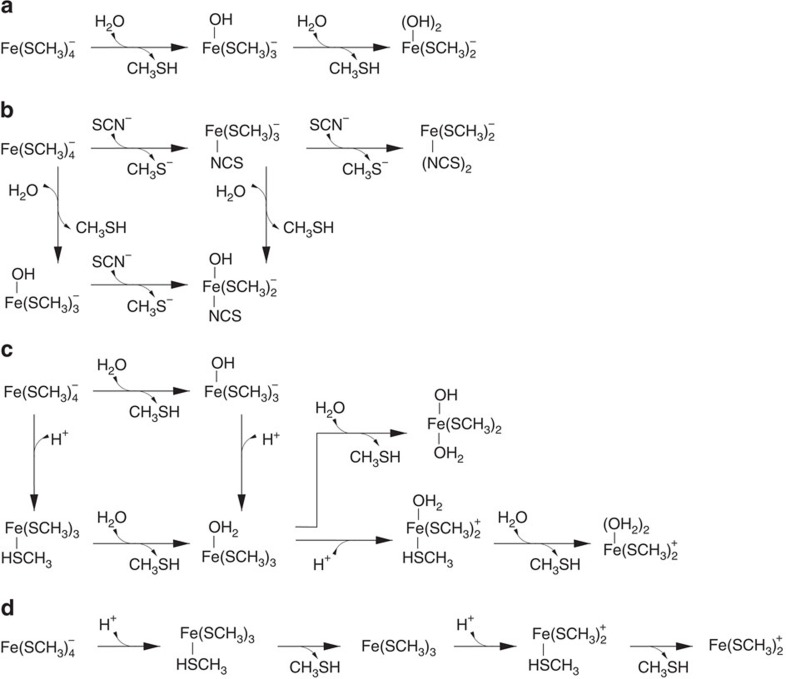
Scheme of the Fe(SCH_3_)_4_^−^ model reactions studied. (**a**) Pure water substitution (reaction A); (**b**) substitution in the presence of SCN^−^ (reaction B); (**c**) water substitution under acid catalysis (reaction C); (**d**) bond dissociation under acid catalysis (reaction D).

Although our AFM experiments allow the observation of ferric–thiolate bond rupture in rubredoxin, they cannot give indications as to the mechanism by which rupture occurs within the protein. This rupture could occur in a heterolytic manner, leading to thiolate anion; it can also be homolytic, resulting in a thiol radical^44^,^45^. In our previous QC studies, we found that the rupture of ferric–thiolate bonds without ligand substitution or protonation follows a homolytic mechanism^30^. To gain insights into the mechanism by which FeS_4_ rupture occurs within the protein in the presence of exogeneous ligands, we used QC calculations based on density functional theory (DFT) to model Fe–S bond cleavage reactions. The Fe(SCH_3_)_4_^−^ compound was chosen to model the ferric–thiolate centre in rubredoxin exposed under mechanical tension. In order to unfold rubredoxin completely, two ferric–thiolate bonds have to be broken. These reactions were investigated under three conditions: pure water, acidic conditions and in the presence of SCN^−^, as shown in Fig. 4a–d. Reactions in Fig. 4a–d are referred to as Reaction A–D, respectively. Calculations were performed in an implicit aqueous solvent to account for the aqueous environment in which AFM experiments were conducted. Further details on the calculation procedures are given in the Methods section.

In pure water and in the presence of SCN^−^, we observed that substitution reactions have significantly lowered activation barriers compared with bare FeS bond dissociation without ligand exchange. Under acidic conditions, both ligand substitution and bare dissociation are competitive. In contrast to our previous study in which the rupture of ferric–thiolate bonds follows a homolytic mechanism^30^, we found that the rupture of ferric–thiolate bonds proceeds via a heterolytic mechanism in the reactions with ligand substitution and leaving-group protonation; no electronic spin crossing is observed, as all products stay in their initial reactant high-spin (sextet) configuration.

Sequential FeS bond cleavage from Fe(SCH_3_)_4_^−^ without ligand exchange will proceed by a simple dissociation mechanism, in which products have a three- and a two-coordinated ferric ion centre (Dn only mechanism, Fig. 4d). On the other hand, ligand substitution reactions may proceed through three possible mechanisms: (A) initial thiol(ate) dissociation, which results in a stable intermediate with three-coordinated iron, followed by the new ligand addition (Dn+An mechanism); (B) ligand addition forming a stable penta-coordinated intermediate, followed by thiol(ate) dissociation (An+Dn mechanism); (C) simultaneous ligand addition and thiol(ate) elimination without a stable intermediate (AnDn mechanism).

**Figure 5:**
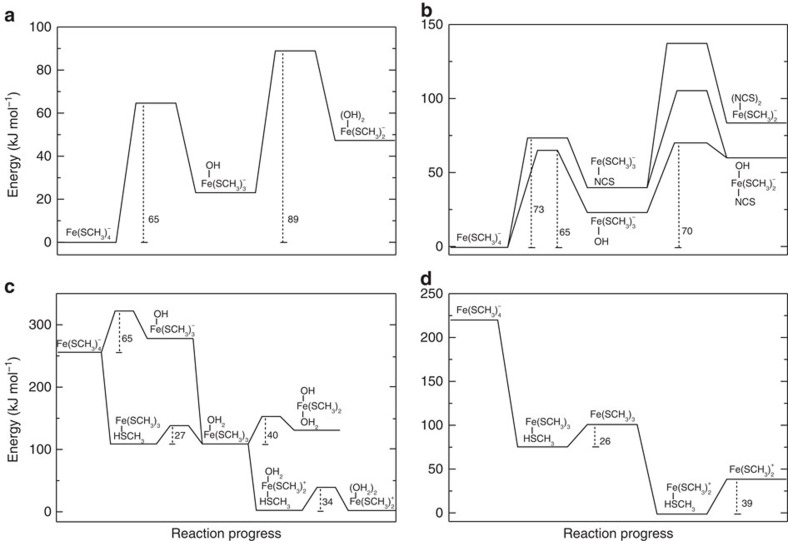
Relative energy profiles indicate possible mechanisms for the ferric–thiolate bond cleavage. Calculations were performed at the DFT level of theory in implicit aqueous solvent for the Fe(SCH_3_)_4_^−^ model reactions in their sextet electronic spin states. (**a**) Pure water substitution; (**b**) substitution in the presence of SCN^−^; (**c**) water substitution under acid catalysis; (**d**) bond dissociation under acid catalysis.

For reactions occurring in pure water (Fig. 4a), the two ligand substitution reactions proceed via an AnDn mechanism with a concerted proton transfer from the attacking water to the thiolate-leaving group. Two-dimensional potential energy scans were conducted to determine the concerted proton and ferric ion transfer reaction barrier. Figure 5a shows that the second substitution reaction is rate-limiting, with a barrier of 89 kJ mol^−1^.

For the reaction in the presence of SCN^−^, we investigated the sequential substitution of two SCN^−^ towards iron and the substitution of one SCN^−^ and one water molecule (Fig. 4b). Figure 5b shows that the reaction profile with the lowest barrier corresponds to an initial reaction with water (barrier of 65 kJ mol^−1^) followed by the rate-limiting substitution with SCN^−^, with a barrier of 70 kJ mol^−1^, corroborating the first-order character of the SCN^−^ reaction. The mechanism for SCN^−^ substitution towards the ferric ion centre in either Fe(SCH_3_)_4_^−^ or Fe(SCH_3_)_3_OH^−^ is An+Dn, with a shallow and transient penta-coordinated intermediate. The reaction barriers are much higher for two sequential SCN^−^ reactions, and also for the dissociative mechanisms (Dn+An and Dn only, data not shown).

For the reactions under acidic conditions, protonation of the thiolate ligand leads to considerably lower barriers for FeS bond rupture. Figure 5c shows that the second substitution reaction is rate-limiting, with a barrier of 34 kJ mol^−1^, corresponding to the water addition step in a Dn+An mechanism. A competitive channel with a barrier of 40 kJ mol^−1^ was found for water substitution without acid catalysis in the second FeS bond cleavage, corresponding to thiol dissociation via an An+Dn mechanism. A purely dissociative mechanism without ligand substitution at iron (Dn only, Fig. 4d) also becomes competitive, with a barrier of 39 kJ mol^−1^ (Fig. 5d), corresponding to the second FeS bond dissociation. It should be noted that for the purely dissociative mechanism (Dn only) shown in Figs 4d and 5d, no transition state was identified, and barriers correspond to the dissociated product energies. Thus, based only on the calculated energy profiles, we are unable to discriminate between reaction C and D or between the associative and dissociative mechanisms under acidic conditions.

In order to resolve this uncertainty and directly probe the effect of mechanical force on the ferric–thiolate bond dissociation, we employed the constrained geometries simulate external force (COGEF) method and determined the rupture or maximum force along the dissociation profiles (Supplementary Fig. 3). Table 1 shows results for the reactions in pure water and acidic conditions. The calculated forces are consistently about twice as high as the average of the measured AFM rupture forces. This shift between calculated COGEF and measured forces has been observed for other mechanochemical activated reactions^36^.

Most importantly, we observe a clear decrease in the calculated maximum force when going from the pure water (*F*_max_=651 pN) to the acid-catalysed reaction (*F*_max_=350 pN), in agreement with the AFM data. Comparison of the calculated rupture forces helps to clarify the mechanochemical mechanism of ferric–thiolate bond dissociation under acidic conditions. The mechanism with the lowest rupture forces corresponds to reaction C with a concerted AnDn mechanism in the first FeS bond cleavage (*F*_max_=298 pN and barrier of 27 kJ mol^−1^) and a protonated intermediate that dissociates via a Dn+An mechanism in the second FeS bond cleavage (*F*_max_=350 pN and barrier of 34 kJ mol^−1^). Reaction D and the two deprotonated steps in reaction C have significantly higher calculated rupture forces (Table 1).

On going from the pure water to the acid-catalysed reaction, the change in reaction coordinate between bound and transition states estimated from the AFM experiments (Δ*x*_u_) increases by 0.6 Å. This is comparable to the 0.3 Å increase obtained from the calculated differences of ferric–thiolate bond distance between reactant and transition state structures (Δ*d*(FeS) in Table 1) for the reactions with the lowest rupture force. Differences between the experimental and calculated values for Δ*x*_u_ and rupture force (*F*_max_) may be attributed to approximations in the QC level of theory (DFT) and to a lack of description of the full protein structure in the calculated model.

## Discussion

The chemical reactivity of metal centres in metalloproteins plays critical roles in the functioning of metalloproteins in biology. In physiological environments, metal centres of many metalloproteins are often enclosed by the 3D structure of proteins, making it difficult to investigate the reactivity of metal centres in aqueous environments. Synthetic inorganic analogues provide excellent model compounds for probing the structure and function of metal centres in metalloproteins. However, most inorganic analogues, including iron–sulfur clusters, are labile towards water and/or oxygen, in the absence of the protective 3D structure of proteins. As a result, many experiments using inorganic analogues have been conducted in non-aqueous organic solvent, with only a few recent studies performed with partially protic solvents^43^. Because of this, it remains challenging to investigate the chemical reactivity of metal centres in metalloproteins in aqueous environments in the presence of proteins' 3D structure.

In this paper, using rubredoxin as a model system, we have demonstrated the utility of single-molecule force spectroscopy techniques in probing the chemical reactivity of metal centres in metalloproteins in aqueous solution. Two different chemical reactions, protonation and ligand exchange reaction, were observed to occur at the FeS_4_ centre of rubredoxin using AFM. Our results showed that the bond strength of ferric–thiolate bonds in rubredoxin is weakened and their lifetime shortened when competing interactions with exogeneous ligands are present. These results are corroborated by QC calculations, and demonstrate that partial unravelling of rubredoxin facilitates the attack of the FeS_4_ centre by exogeneous ligands in aqueous solution, even though most of the proteins' 3D structure remains largely intact. Moreover, QC calculations reveal that ferric–thiolate bond rupture follows a heterolytic reaction mechanism in the presence of ligands, which is different from the homolytic rupture of ferric–thiolate bonds without ligand substitution or protonation. It is worth noting that heterolytic reaction mechanisms are also common for other reactions in solution. For example, it has been observed in QC calculations that the mechanical rupture of the backbone of polyethylene glycol in solution follows a heterolytic reaction mechanism^44^.

Compared with chemical denaturation methods (which result in complete unfolding of the metalloprotein), the force spectroscopy method demonstrated here allows one to partially unfold a metalloprotein to expose the metal centre while keeping the rest of protein structure largely intact, thus enabling the examination of the chemical reactivity of metal centres in the presence of protein 3D structure in aqueous solution. It is worth noting that how the metal centre becomes exposed is different in mechanical and chemical denaturations. Thus, the chemical reactivity of metal centres may exhibit differences in mechanical and chemical denaturation studies. Nonetheless, the force spectroscopy method demonstrated here provides a new approach to study the chemical reactivity of metal centres in metalloproteins, which will complement other methods (including chemical denaturation and synthetic analogues) and provide additional insights into this important problem. Although this study is carried out on the model protein rubredoxin, the extension of the novel method demonstrated here to other proteins is currently under investigation in our laboratories. In principle, the method proposed should be applicable to the study of a wide variety of other metalloproteins in aqueous solution, including such complex examples as the nitrogenases and hydrogenases. Moreover, the forced unfolding that we employ may be able to mimic the physiological conditions to which some metalloproteins are subject *in vivo*, and thus help elucidate their chemical reactivity and functions in a setting that is close to that *in vivo*.

## Methods

### Protein engineering

The gene encoding protein chimera RD-GB1 was constructed as previously reported^26^,^46^. The protein was expressed using pQE80L vector in *Escherichia coli* strain DH5α and purified with Co^2+^-affinity chromatography using TALON His-Tag purification resins (Clontech), followed by ion exchange chromatography using a Mono Q 5/50 anion exchange column (GE Healthcare). The resultant ferric-form rubredoxin chimera was reacted with BM(PEO)_3_ (1, 8-bis (maleimido)triethylene glycol, Molecular Biosciences) through a thiol–maleimide coupling reaction, forming the polyprotein (RD-GB1)_*n*_.(ref. 46). The specific rubredoxin we studied is from the hyperthermerophile *Pyrococcus furious*. The ferrous rubredoxin (with an oxidation state of +2) is air-sensitive because of oxidation; however, the ferric form we studied here is stable in air. In our experiment, the protein is kept in Tris buffer under ambient conditions.

### Single-molecule AFM experiments

Single-molecule AFM experiments were carried out on a custom-built AFM as reported previously^34^. Before each experiment, each Si_3_N_4_ cantilever (Bruker Corp) was calibrated in solution using the equipartition theorem (with typical value of ∼40 pN nm^−1^). In a typical AFM stretching experiment, the polyprotein sample (2 μl, 2 mg ml^−1^) was added on a clean glass coverslip covered by Tris buffer (∼50 μl, pH 7.4, 100 mM Tris and NaCl). The protein was allowed to absorb on the coverslip for ∼5 min before the experiment. Concentrations of 5, 50, 500 and 800 mM of KSCN were obtained by adding an appropriate quantity of 1 M KSCN stock solution to the Tris buffer.

### QC calculations

DFT with the unrestricted B3LYP functional^47^,^48^ and the 6-31+G(2df,p) Pople basis set^49^ were used for quantum chemical calculations. The minimum energy pathway for FeS bond dissociation obtained with this level of theory compares satisfactorily to the energy profile obtained with high-level *ab initio* multiconfigurational calculations (data not shown). Electronic structure calculations were performed with GAUSSIAN 2009 revision A.01 (ref. 50). FeS bond dissociation and FeO (water) or FeN (thiocyanate) bond formation were mimicked by optimizing the complex with a fixed breaking or forming bond distance, respectively. The relevant intermediate structures were then optimized without constraints. Transition states were not fully optimized; however, energy barriers correspond to the highest-energy structures found along the fixed bond scans. Energy differences between these structures and actual transition states were smaller than ∼2 kJ mol^−1^. Geometry optimizations of concerted iron and proton transfer reactions were conducted by additional constraints in the hydrogen-acceptor bond distance, using the pDynamo library^51^ interfaced with the ORCA programme version 3.01 (ref. 52). Calculations were conducted in aqueous solvent using the Polarizable Continuum Model^53^ (PCM, in GAUSSIAN), or the Conductor-like Screening Model^54^ (COSMO, in ORCA). The rupture force or the maximum force along the FeS dissociation pathway was calculated with the constrained geometries simulate external force (COGEF) method^45^,^55^. The distance between two hydrogen atoms from different methyl groups were constrained (COGEF extension) and the remaining degrees of freedom were optimized. Force versus extension curves were obtained by finite differences of the energy versus extension profiles after cubic spline fitting. All relative energies reported here contain electronic, nuclear repulsion and solvent contributions only.

The pH dependence of the electrostatic free energy between the folded and unfolded states of rubredoxin (Supplementary Fig. 4) was estimated using Poisson–Boltzmann calculations using the standard method proposed in ref. 56.

## Additional information

**How to cite this article:** Zheng, P. *et al*. Force-induced chemical reactions on the metal centre in a single metalloprotein molecule. *Nat. Commun*. 6:7569 doi: 10.1038/ncomms8569 (2015).

## Supplementary Material

Supplementary Information Supplementary Figures 1-4

## Figures and Tables

**Table 1:** Rupture force and difference in bond distance between reactant and transition state.

Reaction	Mechanism	*F*_max_ (pN)	Δ*d*(FeS) (Å)
A, step 1	AnDn	548	0.2
A, step 2	AnDn	651	0.3
C, step 1 protonated	AnDn	298	0.2
C, step 2 neutral	An+Dn	963	0.2
C, step 2 protonated	Dn+An	350	0.6
D, step 1	Dn	579	1.1

Rupture force (*F*max in pN) and difference in bond distance (Δ*d*(FeS) in Å) are calculated with the COGEF method. Steps 1 and 2 correspond to the first and second FeS bond cleavage, respectively (Fig. 4).
