# Supplementary material for: Force-induced chemical reactions on the metal centre in a single metalloprotein molecule
Source: Nat Commun. 2015 Jun 25;6:7569. doi: 10.1038/ncomms8569 (PMC4491811; doi:10.1038/ncomms8569)
Supplement: Supplementary Information — Supplementary Figures 1-4 [file ncomms8569-s1.pdf]

# Supplementary Information

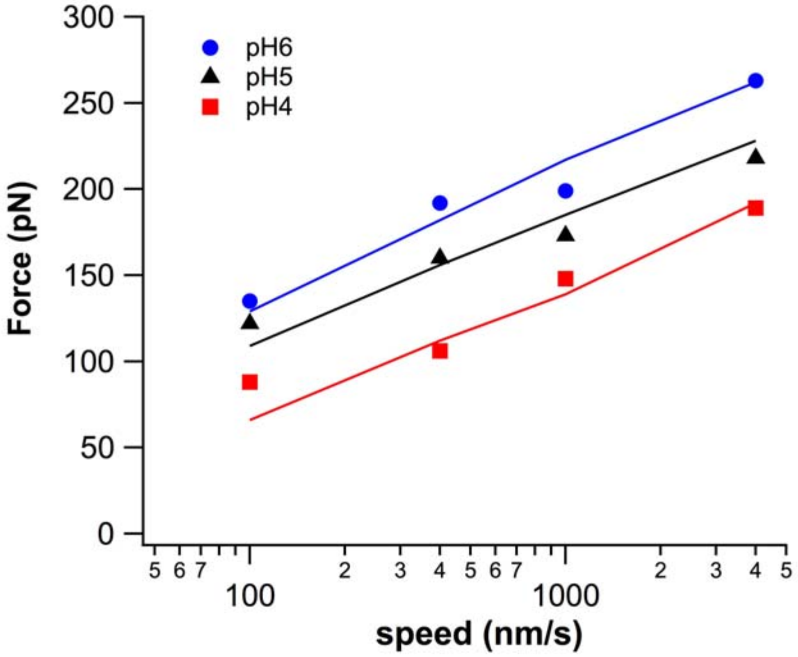


Supplementary Figure 1. Pulling speed dependence of the unfolding force of rubredoxin at pH 4, 5, and 6.

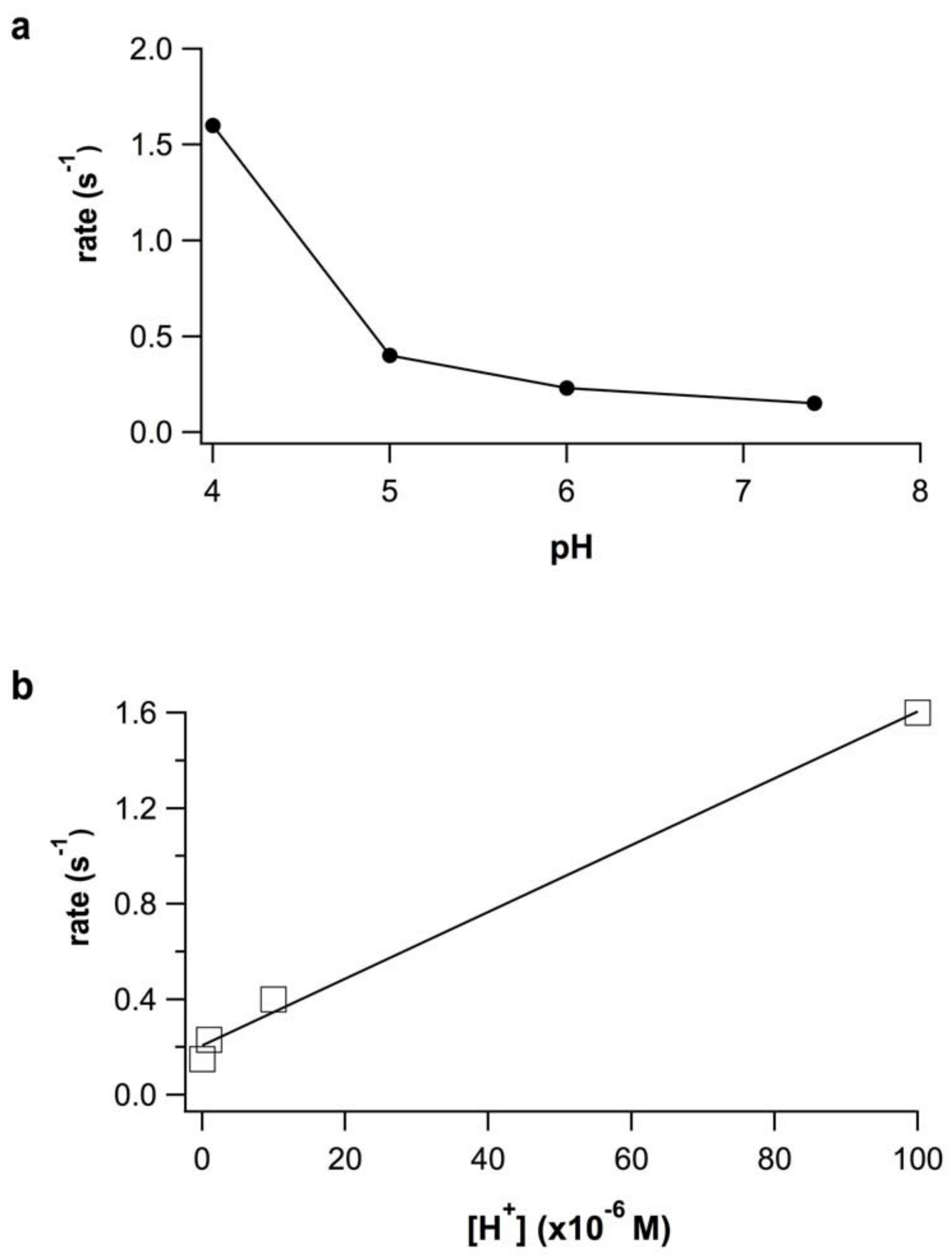


Supplementary Figure 2. Spontaneous dissociation rate $\alpha_0$ of $FeS_4$ center depends on pH. A) $\alpha_0$ versus *pH*. B) $\alpha_0$ versus $[H^+]$. $\alpha_0$ appears to be linearly dependent upon $[H^+]$. The solid line is a linear fit to the experimental data. However, we leave the validation of the precise relationship between $\alpha_0$ and [H+] to future systematic experiments.

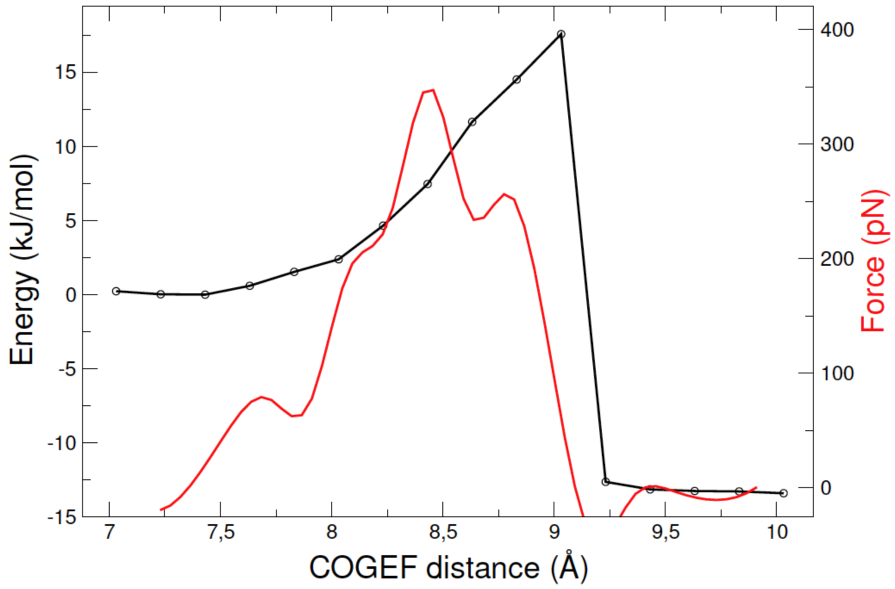


Supplementary Figure 3. Relative energy and force profiles calculated at the DFT level of theory in implicit aqueous solvent with the constrained geometries simulate external force (COGEF) method for the dissociative step in the second FeS bond cleavage in acidid media (Reaction C, step 2 protonated in Figure 4). The rupture force ($F_{max}$) is observed at 8.4 Å and the FeS bond is broken at 9.2 Å.

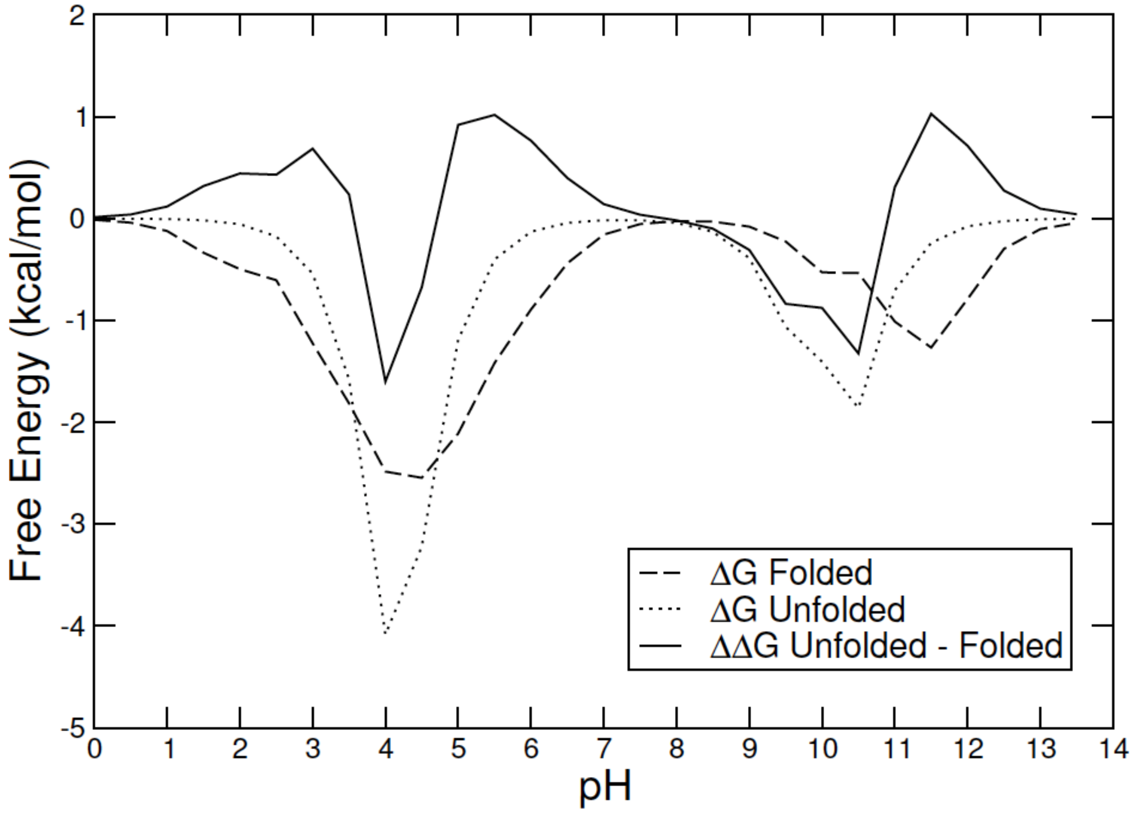


Supplementary Figure 4. The pH-dependence of free energy between folded and unfolded states of rubredoxin obtained with Poisson-Boltzmann electrostatics calculations. The variation in the free energy difference between folded and unfolded forms as a function of pH is quite small (± ~ 1.0 kcal $mol^{-1}$) and there is no systematic trend with pH.The result shows small changes in polypeptide stability and suggests that protonation of the FeS center, instead of the peptide chain, alters the force-extension curves.
